# First phenotypic description of a female patient with c.610 T > C variant of *GLA*: a renal-predominant presentation of Fabry disease

**DOI:** 10.1186/s12881-020-01071-5

**Published:** 2020-06-26

**Authors:** Sophie Greillier, Laurent Daniel, Catherine Caillaud, Bertrand Dussol, Guy Touchard, Jean-Michel Goujon, Noémie Jourde-Chiche, Mickaël Bobot

**Affiliations:** 1 AP-HM, Centre de Néphrologie et Transplantation Rénale, CHU de la Conception, AP-HM, Marseille, France; 2 Aix-Marseille Univ, C2VN, INSERM, INRAE, Marseille, France; 3 AP-HM, Laboratoire d’Anatomie Pathologique, CHU de la Timone, Marseille, France; 4 Laboratoire de Biochimie, Métabolomique et Protéomique, AP-HP. Centre-Université de Paris, Hôpital Necker-Enfants Malades, Paris, France; 5 Laboratoire d’Anatomie Pathologique, CHU de Poitiers, Poitiers, France

**Keywords:** Fabry disease, Female, Phenotype, *GLA* variant, lysoGb3, Renal involvement

## Abstract

**Background:**

Fabry disease (FD) is an X-linked lysosomal storage disorder due to deficient alpha-galactosidase activity leading to intracellular glycosphingolipid accumulation. Multiple variants have been reported in the *GLA* gene coding for alpha-galactosidase, and the question of the pathogenicity of rare variants needs to be addressed, especially in patients with mild phenotypes.

**Case presentation:**

The patient, a 37-year-old female, presented with a persistent proteinuria after an otherwise uncomplicated first pregnancy. Renal biopsy showed both mild mesangial IgA deposits, and a striking vacuolization of podocytes and tubular cells consistent with Fabry disease. On electron microscopy, discrete but characteristic pseudo-myelinic lamellar inclusions were observed in the podocytes’ lysosomes. A more detailed physical examination revealed an angiokeratoma, and medical history ancient acroparesthesia. There was no cardiac or cerebral involvement of Fabry disease on magnetic resonance imaging. While blood enzymatic activity of alpha-ga lactosidase was normal in this patient, lysoGb3 was elevated (3 N), and a rare heterozygous variant called c.610 T > C was documented in *GLA* gene. The patient was treated with an ACE inhibitor, with a rapid decrease in proteinuria. After a 5-year follow-up, her renal function has remained normal, with mild proteinuria, and normal cardiac echography.

**Conclusions:**

We report and phenotypically describe the first case of a Fabry disease female patient carrying the *GLA* c.610 T > C variant associated with a renal-predominant clinical presentation.

## Background

Fabry disease (FD) is a rare systemic storage disorder resulting from a deficiency of lysosomal alpha-galactosidase A, which leads to the accumulation of sphingolipids (mainly globotriaosylceramide, Gb3) in endothelial cells and various tissues. The enzymatic deficiency is linked to pathogenic variants in the *GLA* gene, located on the X chromosome. The most frequently affected organs are the heart, kidneys, skin, gut and central nervous system [1].

In the “classical” form of FD, typically affecting hemizygous male patients, the first symptoms appear in childhood and associate acroparesthesia, fever, abdominal pain and arthralgia. The cardiac, neurologic and renal manifestations generally appear between the fourth and sixth decade with potentially serious consequences: hypertrophic cardiomyopathy leading to heart failure, end-stage renal disease, and strokes with severe neurological sequelae [2].

The severity of clinical presentation is usually correlated with the depth of enzyme deficiency (alpha-galactosidase activity, AGAL-A) [3], and symptoms in females, who are heterozygous, are usually less pronounced than in males [4]. However, the random silencing of one X chromosome in each cell of female carriers, called “X-chromosome inactivation” or “lyonization”, leads to the sole expression of the genes from the other X chromosome. The proportion of cells in which the normal X chromosome is silenced is not always 50% (a phenomenon called “X-inactivation skewing”), and can vary among the different organs, which results in the various levels of enzymatic deficiency and the broad range of phenotypic patterns observed in females with FD [5, 6].

Moreover, several *GLA* variants are associated with residual enzyme activity in vitro, without typical symptoms of FD (“non-classical” FD) [7], and many recently identified *GLA* variants have undetermined clinical significance [6].

Here we describe the first case of a female patient with a rare *GLA* variant associated with a mild FD phenotype comprising proteinuria, acroparesthesia and angiokeratoma. We detail the renal pathological lesions observed, and discuss the pathogenicity of this *GLA* variant.

## Case presentation

A 37-year-old female, native of Romania, was referred to our centre for evaluation of persistent albuminuria (1 g per day) after an otherwise uncomplicated first pregnancy, with normal kidney function (serum creatinine 65 μmol/L, estimated glomerular filtration rate (eGFR) 94 mL/min/1.73 m^2^ with the CKD-Epi formula).

**FIG. 1:**
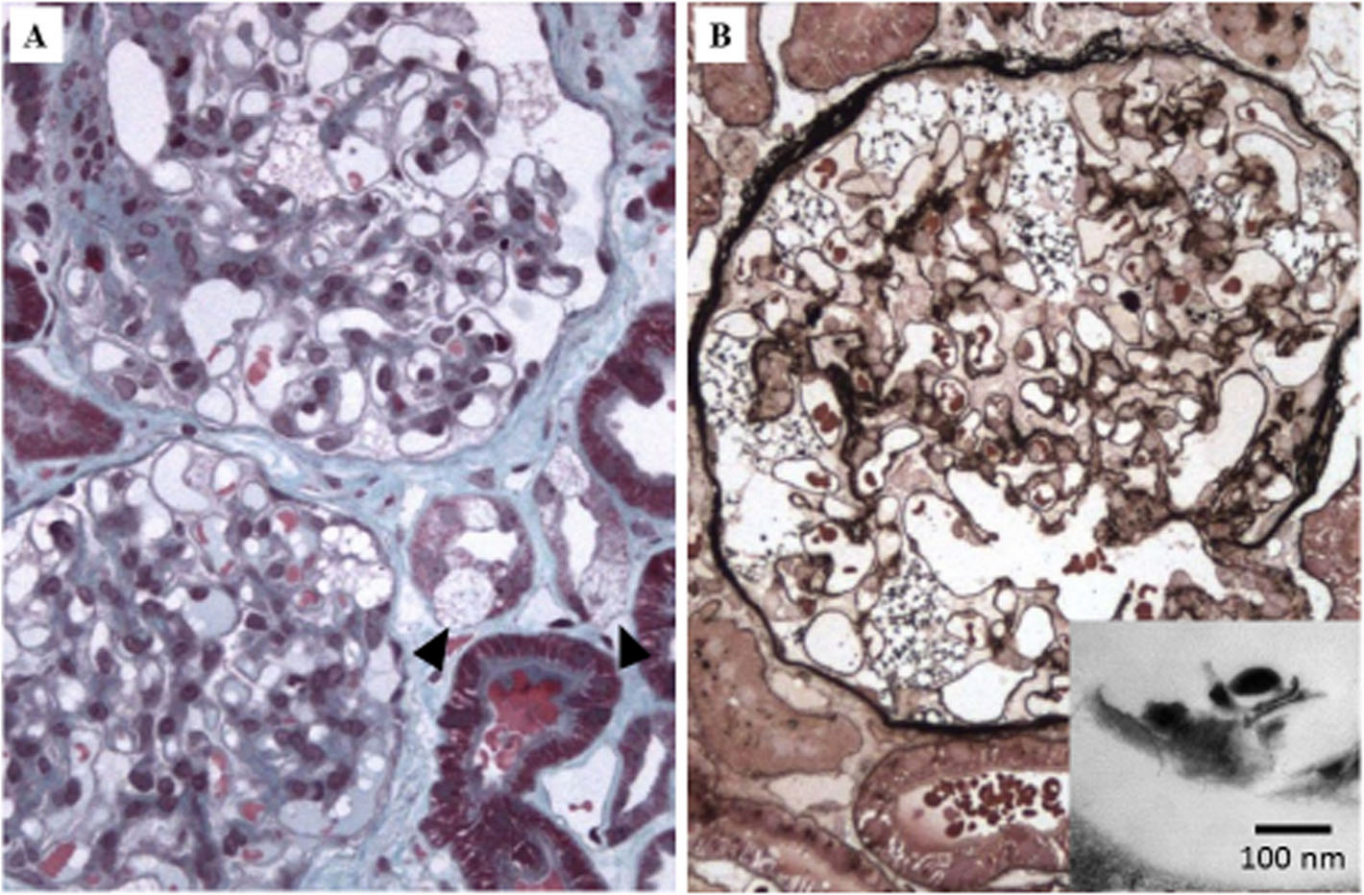
**a:** Enlarged podocytes with bubbly cytoplasm associated with involvement of few distal tubular cells (arrowheads). Masson’s trichrome, × 200. **b:** Massive vacuolization of podocytes without endocapillary alterations. Jones silver staining, × 400. **Inset:** electron microscopic. Vacuoles are empty or contain few simplified pseudo-myelin strips without zebra bodies

Kidney biopsy showed mild mesangial IgA deposits (+) in immunofluorescence, without mesangial proliferation, as well as extensive vacuolisation of the podocytes and tubular epithelial cells, suggestive of FD [8]. In electron microscopy, cytoplasmic vacuoles were observed in the podocytes and tubules, with discrete pseudomyelinic figures **(**Fig. 1**).**

The patient’s medical history was as follows: chronic gastritis, chronic hand pain, a posteriori characterized as neuropathic (acroparesthesia). A detailed skin examination revealed an angiokeratoma confirmed by biopsy. There was no obvious family history of kidney disease or FD, but the family could not be thoroughly explored.

Further investigations did not reveal any other organ involvement of FD: normal sudation, normal ophthalmologic examination, normal cardiac explorations (ECG, echocardiography, cardiac magnetic resonance imaging (MRI)), and normal cerebral MRI.

There was no leukocyte enzyme deficiency (AGAL-A was 70% of female normal controls, reference range: 104–236 nmol/h/mg protein), but an elevated level of lyso-Globotriaosylsphingosine (lyso-Gb3) was detected by tandem mass spectrometry on dried blood (10 ng/mL, normal 0–3.5).

Sanger sequencing based on the analysis of the seven *GLA* exons including exon-intron junctions revealed a heterozygous variant within exon 4 of the *GLA* gene (reference sequence: NM_000169.2): c.610 T > C or p.(Trp204Arg) (Supplementary Figure **1****)**. This variant can be considered as likely pathogenic (class 4) according to the ACMG variant classification: PS3 (functional studies performed by Lukas et al.) [9], PM2 (absent in population databases), PP3 (multiple lines of computational evidence supporting a deleterious effect). X-inactivation study was not performed (not routinely done in our country).

Since the clinical presentation was not severe, with normal enzyme level and proteinuria rapidly decreasing with an angiotensin converting enzyme inhibitor, the patient was offered two therapeutic options: early enzyme replacement therapy (ERT), or regular monitoring. She chose monitoring.

After a 5-year follow-up, the patient’s kidney function remained normal (serum creatinine 70 μmol/L, eGFR 87 ml/min/1.73m^2^) with normal blood pressure and mild proteinuria (urinary protein/creatinine ratio 0.3 g/g). Annual echocardiography shows no myocardial hypertrophy. The patient’s 5-year-old son, who is asymptomatic, without proteinuria, has not yet been tested for enzyme activity or genetic explorations and is monitored by paediatricians.

## Discussion and conclusions

We report here the case of a female patient with a rare *GLA* variant (c.610 T > C) with elevated lysoGb3 despite a normal AGAL-A activity, sphingolipid accumulation in podocytes and tubular epithelial cells, angiokeratoma and neuropathic pain. According to FD definition criteria [7, 10], this patient suffers from a classical form of FD, with typical symptoms (neuropathic pains, angiokeratoma) and a documented variant of the *GLA* gene. However, this heterozygous variant has never been described in a female patient before.

The same variant was reported only once, in a series of new *GLA* variants, in a male hemizygous patient [9]. This patient was a carrier of the same c.610 T > C variant, with elevated blood levels of lyso-Gb3, but unlike our patient, no AGAL-A activity was detected in vitro. No clinical data were available for this case. In addition to the 0% enzymatic activity, the deleterious effect of the c.610 T > C variant is supported by predictive tools such as Poly Phen–2 (probably damaging, score 1) or SIFT (not tolerated) [9, 11]. This variant has not been reported in the GnomAD database, but 3 occurrences can be found in ClinVar: one without clinical information, and two related to the case discussed above (Lukas et al. 2014) [9]. This is therefore the first c.610 T > C variant with a detailed phenotypic description, and the first case in a female patient.

The discrepancy observed in our female patient between the normal leucocyte enzyme activity in vitro and the elevated lyso-Gb3 with localized manifestations of FD is possibly related to a skewed X-chromosome inactivation (lyonization) [5], with a patchy AGAL-A defect and glycosphingolipid accumulation within tissues [4].

Since the broader screening for non-classical FD in patients with late symptomatology or single organ involvement (cardiac, renal, cerebrovascular variants of FD) [12], many *GLA* variants of unknown significance have been reported [9]. These variants can be associated with classical FD, attenuated or non-classical FD, or absence of disease [7, 10]. In this context, and in asymptomatic female patients with normal AGAL-A [13] the lyso-Gb3 assay is of particular interest [14]: elevated lyso-Gb3 is highly suggestive of classical FD if a new variant is discovered without typical FD symptoms [15].

In this respect, the renal involvement of this patient is both highly suggestive of FD (vacuolisation with pseudomyelinic bodies) and atypical (additional IgA mesangial deposits). The patient had never been treated with medications associated with pseudomyelinic inclusions (such as antimalarials) [16–19] or with lyso-Gb3 elevation (such as amiodarone) [19].

In this form of FD, with mild symptoms and normal AGAL-A, the benefit of enzyme replacement therapy (ERT) remains uncertain. ERT significantly reduces plasma lyso-Gb3 [8] and pain scores [20]. A task force of the American College of Medical Genetics suggests that ERT should be initiated when organ damage is evident and at the discretion of the clinician in asymptomatic hemizygotes, considering family history [21]. Hopkin et al. recommended initiating ERT in symptomatic patients of any age or sex, including patients with evidence of tissue damage on kidney biopsy; the decision is left to the clinician for asymptomatic patients [22]. The European Consensus distinguishes between male and female patients and between classical and non-classical forms of the disease: for males with classical FD, ERT should be initiated from the age of 16 years; males with non-classical FD and females with classical FD should be treated at the onset of clinical symptoms; ERT may be considered in females with non-classical FD and clinical signs [23]. However early treatment of patients with no or mild symptoms remains controversial [7].

Another treatment option in FD is the administration of a pharmacological chaperone, such as oral migalastat, to reduce the degradation of misfolded alpha-galactosidase and promote its trafficking from the endoplasmic reticulum to the lysosomes, in order to increase its residual lysosomal activity [24]. However, not all mutated enzymes are migalastat-amenable, since the mutated enzyme must have retained its catalytic activity despite abnormal folding [25]. According to predictive models described elsewhere [9, 26], no benefits were expected from the pharmacological chaperone with the current variant.

A follow-up strategy was therefore proposed and chosen for this patient, with no worsening of the renal phenotype and no new organ damage after 5 years. This is still insufficient to conclude on the long-term risk of worsening chronic kidney disease or extra-renal damage, and long-term annual follow-up is planned for this patient.

In conclusion, we described the first c.610 T > C *GLA* variant with a detailed phenotypic description of a renal-predominant presentation of Fabry disease, and the first case in a female patient.

## Supplementary information

**Additional file 1.**

## Data Availability

The datasets generated and analysed during the current study and the raw sequence data obtained as a result of Sanger sequencing **(Supplementary Figure** **1****)** are available in the NCBI ClinVar repository [Accession number: SCV001250894; https://www.ncbi.nlm.nih.gov/clinvar/variation/217390/] and in NCBI dbSNP repository [https://www.ncbi.nlm.nih.gov/snp/rs869312148]. The reference GLA sequence is available in the GenBank repository [Accession number: NM_000169; https://www.ncbi.nlm.nih.gov/nuccore/NM_000169].

## Abbreviations

AGAL-A: Alpha-galactosidase activity
eGFR: Estimated glomerular filtration rate
ERT: Enzyme replacement therapy
FD: Fabry disease
Gb3: Globotriaosylceramide
MRI: Magnetic resonance imaging
