# Supplementary figures and images for: First phenotypic description of a female patient with c.610 T > C variant of GLA: a renal-predominant presentation of Fabry disease
Source: BMC Med Genet. 2020 Jun 26;21:137. doi: 10.1186/s12881-020-01071-5 (PMC7320597; doi:10.1186/s12881-020-01071-5)

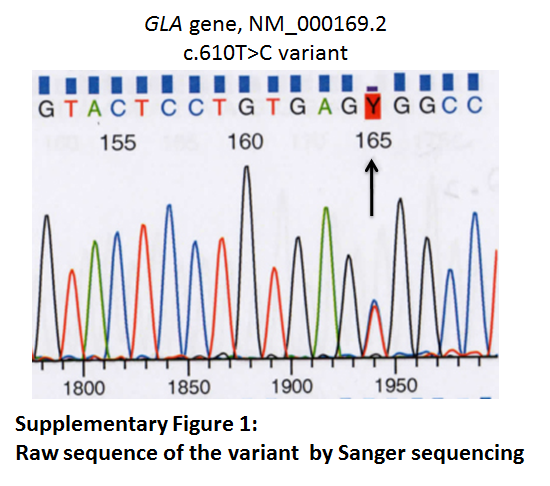

Supplement: Supplementary file 1 — Additional file 1. [file 12881_2020_1071_MOESM1_ESM.tif]
